# Recombinant N-acyl homoserine lactone-Lactonase AiiA_QSI-1_ Attenuates *Aeromonas hydrophila* Virulence Factors, Biofilm Formation and Reduces Mortality in *Crucian Carp*

**DOI:** 10.3390/md17090499

**Published:** 2019-08-27

**Authors:** Bao Zhang, Xiyi Zhuang, Liyun Guo, Robert J. C. McLean, Weihua Chu

**Affiliations:** 1 Department of Pharmaceutical Microbiology, School of Life Science and Technology, China Pharmaceutical University, Nanjing 210009, China; 2 Department of Microbiology, Nanjing Institute of Fisheries Science, Nanjing 210036, China; 3 Department of Biology, Texas State University, San Marcos, TX 78666, USA

**Keywords:** N-acyl homoserine lactone lactonase, *Aeromonas hydrophila*, *Crucian Carp*, virulence factors, biofilm

## Abstract

Quorum quenching (QQ) is a promising alternative infection-control strategy to antibiotics that controls quorum-regulated virulence without killing the pathogens. *Aeromonas hydrophila* is an opportunistic gram-negative pathogen living in freshwater and marine environments. *A. hydrophila* possesses an N-acyl homoserine lactone (AHL)-based quorum-sensing (QS) system that regulates virulence, so quorum signal-inactivation (i.e., QQ) may represent a new way to combat *A. hydrophila* infection. In this study, an AHL lactonase gene, *aiiA* was cloned from *Bacillus* sp. strain QSI-1 and expressed in *Escherichia coli* strain BL21(DE3). The *A. hydrophila* hexanoyl homoserine lactone (C6-HSL) QS signal molecule was degraded by AiiA_QSI-1_, which resulted in a decrease of bacterial swimming motility, reduction of extracellular protease and hemolysin virulence factors, and inhibited the biofilm formation of *A. hydrophila* YJ-1 in a microtiter assay. In cell culture studies, AiiA_QSI-1_ decreased the ability of *A. hydrophila* adherence to and internalization by *Epithelioma papulosum cyprini* (EPC) cells. During in vivo studies, oral administration of AiiA_QSI-1_ via feed supplementation attenuated *A. hydrophila* infection in *Crucian Carp*. Results from this work indicate that feed supplementation with AiiA_QSI-1_ protein has potential to control *A. hydrophila* aquaculture disease via QQ.

## 1. Introduction

Quorum sensing (QS) is a cell-to-cell communication pathway in microorganisms, in which the expression of a number of genes, often associated with virulence factors and biofilm formation, is controlled via the production and detection of signal molecules in a population density-dependent manner [1,2]. In many Gram negative bacteria, including several aquaculture pathogens, the quorum signal molecules are N-acyl homoserine lactones (AHLs), which are produced by LuxI homologs and detected by LuxR homologs [3,4,5,6]. Disruption of QS is one competition strategy used by microorganisms and higher organisms. QS disruption can occur via interference with quorum signal production or detection (LuxI and LuxR interference), or alternatively disruption of related regulatory networks [7]. Direct inactivation of quorum signal molecules by enzymatic degradation or modification is another strategy used in microbial competition [8,9]. Signal disruption referred to as quorum quenching (QQ) has been described in several biological systems and is being explored as a novel approach to fight human, plant, and animal infections. QQ is also being examined as a mechanism to combat aquaculture-related infections [10,11,12,13].

AHL-degrading enzymes have been found in several bacteria such as *Bacillus* sp., *Pseudomonas* sp., *Microbacterium testaceum, Ochrobactrum* sp.; as well as fungi including *Ascomycota* and *Basidiomycota*. [14]. There are two different classes of AHL-degrading enzymes, AHL lactonases, which cleave the homoserine lactone ring; and AHL acylases, which cleave the acyl side-chain [8]. Previous investigators have cloned and expressed AHL lactonase-encoding genes (*aiiA*) to study the mechanisms and specificity of QQ reactions [15].

*Aeromonas hydrophila* is a gram-negative bacterium found in both freshwater and marine environments. Some strains of *A. hydrophila* cause hemorrhagic septicemia in marine animals, resulting in great economic losses in China every year [16]. *A. hydrophila* expresses several virulence factors, including hemolysins, cytotoxins, lipases, and extracellular proteases as well as biofilm formation, and all of these phenotypes are regulated by AHL-mediated quorum sensing [17]. Previous studies have shown that *Bacillus* sp. strain QSI-1 can produce quorum quenching enzyme (AiiA_QSI-1_), has probiotic properties and also, can decrease the pathogenicity of *A. hydrophila* infection in zebrafish (*Danio rerio*) and Goldfish (*Carassius auratus*) models [18,19]. In this study, we show that the QQ enzyme, obtained via heterologous expression of AiiA_QSI-1_ in *Escherichia coli*, is able to quench AHLs, reduce the expression of several virulence factors of *A. hydrophila*, and can also increase the survival rate of the *Crucian Carp* against experimental *A. hydrophila* infection.

## 2. Results and Discussion

### 2.1. Expression and Purification of Recombinant AHL Lactonase AiiA_QSI-1_

**Figure 1:**
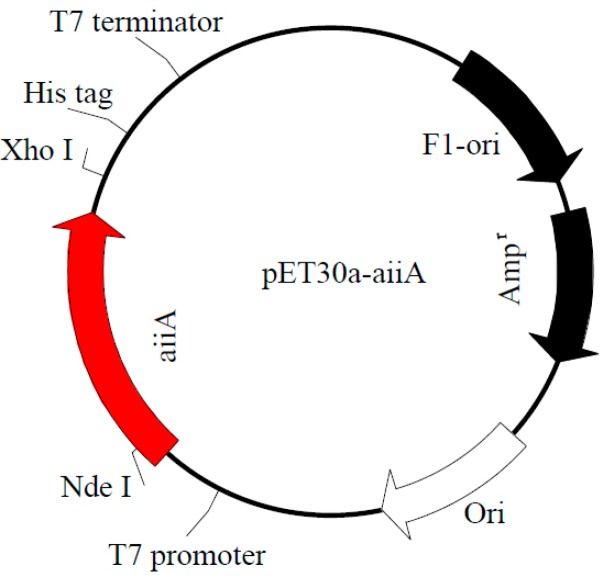
Map of expression plasmid containing aiiA_QSI-1_ gene.

**Figure 2:**
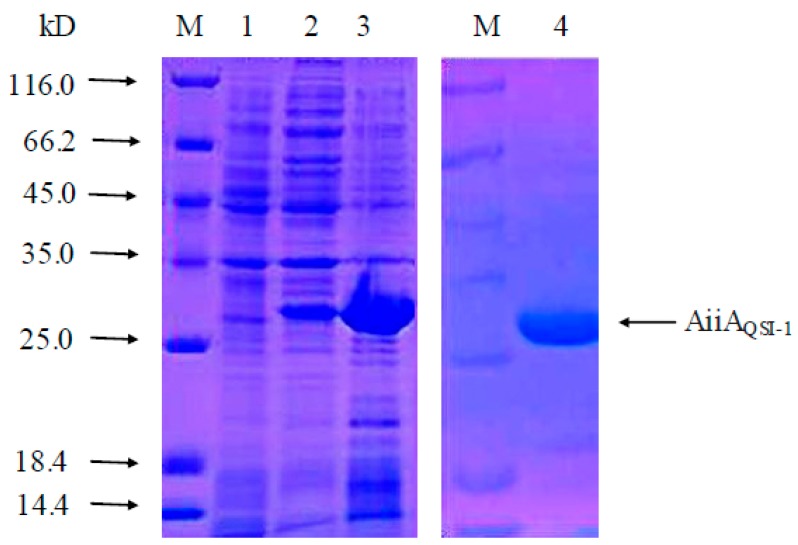
SDS-PAGE analysis of the recombinant AiiA_QSI-1_. M: marker; 1: total protein of noninduced *E. coli* BL21(DE3)pET30a-aiiA_QSI-1_ cell lysate; 2: Supernatant of the sonication product of IPTG-induced *E. coli* BL21(DE3)pET30a-aiiA_QSI-1_; 3: Precipitate of the sonication product of IPTG-induced *E. coli* BL21(DE3)pET30a-aiiA_QSI-1_; 4: purified AiiA_QSI-1_ protein (as indicated by arrow).

In a search using the BLAST program, we found that *aiiA_QSI-1_* gene has 100% similarity in nucleotide sequence with the *aiiA* genes of several species of *Bacillus* such as *B. wiedmannii* and *B. cereus.* The *aiiA_QSI-1_* gene amplified from *Bacillus* sp. strain QSI-1 by PCR, which consists of 747 nucleotides and the gene sequence has been deposited in the GenBank database (MN227141). This gene was then cloned into pET30a(+) vector between Nde I and Xho I restriction sites (Figure 1), which resulted in the expression of the recombinant polypeptide containing 249 amino acids. The transformed *E. coli* strain BL21(DE3) PET30-*aiiA*_QSI-1_ was induced with 0.05 mM IPTG at 37 °C for 4 h. Expressed AiiA_QSI-1_ protein was purified using a Ni-NTA His∙Bind column. SDS-PAGE analysis demonstrated that the purified protein migrated as a single band with a molecular mass about 29 kDa (Figure 2). The concentration of purified protein was 1.5 mg/mL.

**Figure 3:**
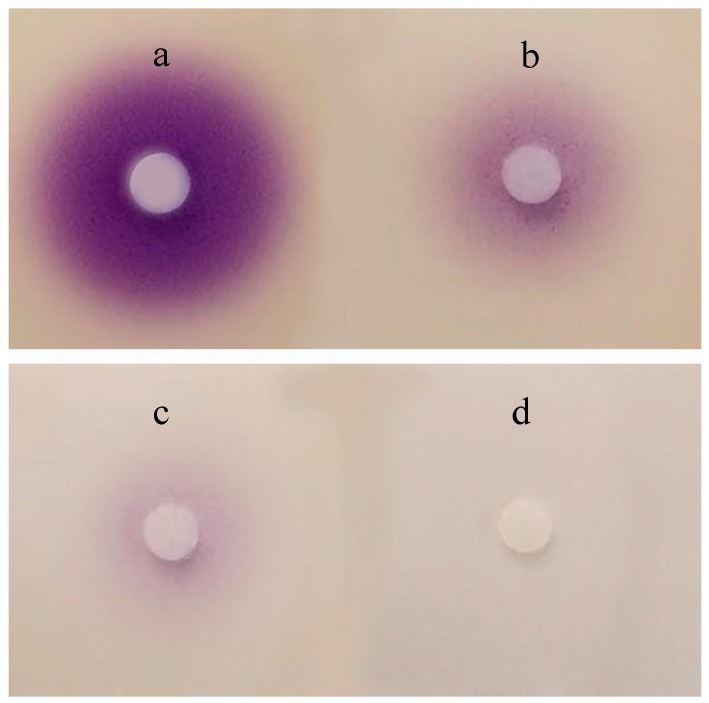
AHL-degrading activity bioassays in vivo. Ten μL C6-HSL (10 μM) was mixed with 0 μL (**a**), 20 μL (**b**), 50 μL (**c**), 100 μL (**d**) AiiA_QSI-1_, respectively and incubated at 30 °C.

The results of the in vitro bioassays suggested that AiiA_QSI-1_ has the ability to degrade N-Hexanoyl-L-homoserine lactone (C_6_-HSL), as indicated by a reduction of the AHL-regulated purple violacein pigment diameter of *Chromobacterium violaceum* CV026 reporter strain (Figure 3).

### 2.2. Effect of AiiA_QSI-1_ on the Motility, Virulence Factors Production and Biofilm Formation in Aeromonas hydrophila

**Figure 4:**
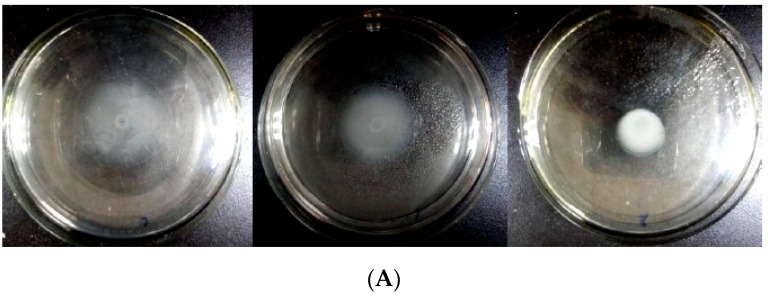

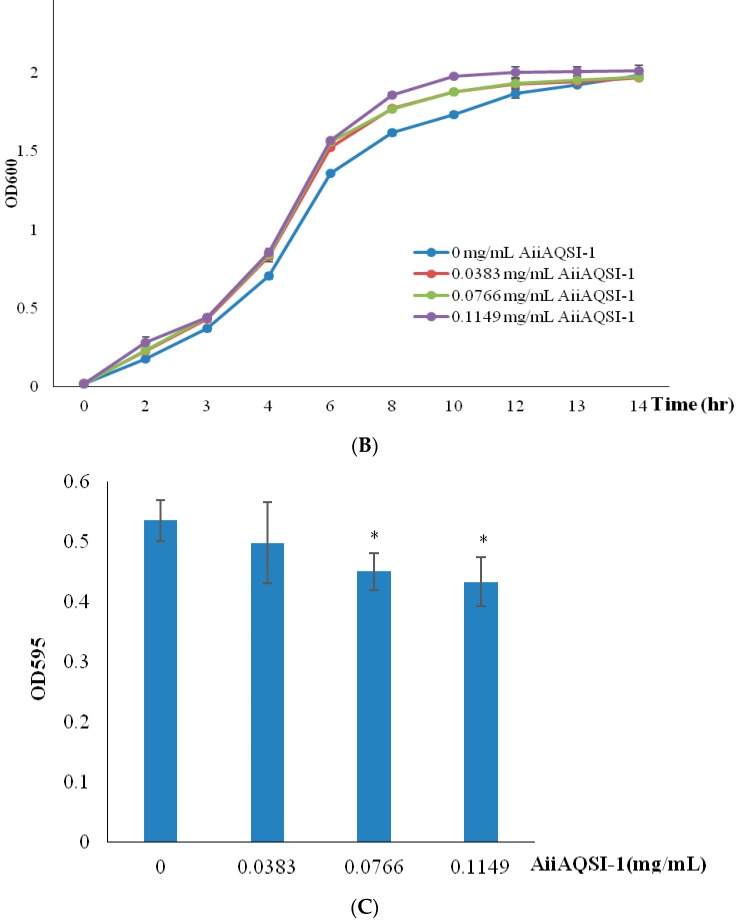
Effect of AiiA_QSI-1_ on the motility, production of virulence factors and biofilm, and growth of *Aerononas hydrophila* YJ-1. Swimming motility (**A**), Growth curve of planktonic bacteria (**B**), and biofilm production (**C**).

In previous studies, we found that *Bacillus* sp. strain QSI-1 was able to inhibit *A. hydrophila* virulence in vitro and in vivo [20]. In order to investigate whether QQ activity in strain QSI-1 was responsible for reduced virulence, we cloned and expressed *aiiA_QSI-1_* gene from strain QSI-1 in *E. coli* BL21(DE3). Our results show that the QQ enzyme AiiA_QSI-1_ obtained by heterologous expression in *E. coli* could exhibit a similar bioprotective effect as the parent *Bacillus* sp. strain QSI-1. Potential anti-QS activity of AiiA_QSI-1_ was investigated on *A. hydrophila* YJ-1 characteristics including swimming motility, production of virulence factors and biofilm formation. As seen in Figure 4, AiiA_QSI-1_ inhibits *A. hydrophila* YJ-1 swimming motility (Figure 4A). The extracellular proteolytic activity and hemolytic activity were also inhibited by AiiA_QSI-1_. Measurements of transparent zones, indicative of proteolytic activity and hemolytic activity, are listed in Table 1. Of note, at the highest AiiA_QSI-1_ concentration tested, 0.1149 mg/mL, extracellular proteolytic activity of *A. hydrophila* YJ-1 was reduced by 66.5% and hemolytic activity reduced by 68.9% when compared to untreated controls. The reduction in virulence was not due to *A. hydrophila* YJ-1 planktonic culture growth inhibition as growth of this pathogen was slightly enhanced by the presence of AiiA_QSI-1_ (Figure 4B). After cultured in the microtiter plates for 36 h, the planktonic bacteria growth has no significant difference with or without AiiA_QSI-1_ treatment. However, *A. hydrophila* YJ-1 biofilm growth in the microtiter assay was reduced at the two highest AiiA_QSI-1_ concentrations tested (Figure 3C).

Our results are in agreement with other in vitro investigations of QQ reduction in virulence in several aquaculture relevant pathogens. Wang et al. [21] demonstrated that AiiA_S1-5_ from *Altererythrobacter* sp. S1-5 showed the QQ efficacy against quorum sensing-mediated virulence traits of *A. hydrophila* and *Vibrio alginolyticus*, such as motility and biofilm formation. Our results also showed AiiA_QSI-1_ can inhibit motility and biofilm formation in *A. hydrophila.* Bai et al. [22] discovered that the over-expressed recombinant AiiA protein from the *aiiA* gene of a *Bacillus thuringiensis* strain BF1, had the ability to inhibit the QS-regulated bioluminescence in *Vibrio harveyi.* Although *V. harveyi* is not considered to be a pathogen, the association of bioluminenscence with QS in this organism is well-documented [23]. These previous studies did not address biofilm formation by *A. hydrophila.* In the present study, we found the AiiA_QSI-1_ can stimulate the planktonic growth of *A. hydrophila* YJ-1 (Figure 3D) but not biofilm formation (Figure 4E). Planktonic growth stimulation may be due to *A. hydrophila* being able to use degraded AHLs or alternatively use the supplemented added AiiA_QSI-1_ proteins as nutrient sources [24] and the biofilm inhibition due to QQ [10]. Another possible reason for the increase of planktonic bacteria during exponential phase maybe due to the AiiA_QSI-1_ protein can inhibit the bacterial motility and decrease the adherence for biofilm formation.

### 2.3. Bacterial Adherence and Internalization to Epithelioma Papulosum Cyprini (EPC) Cells

**Figure 5:**
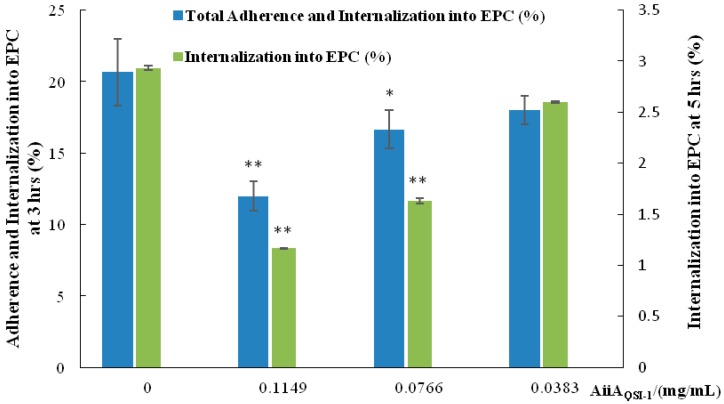
Effect of different concentrations of AiiA_QSI-1_ on *A. hydrophila* YJ-1 adherence and internalization to EPC. * *p* < 0.05; ** *p* < 0.01 in comparison to controls (no added QQ).

Adherence and invasion of *A. hydrophila* YJ-1 to EPC cells were examined under different concentrations of AiiA_QSI-1_ protein. Our study showed that AiiA_QSI-1_ protein exhibited inhibitory activity in both adherence and internalization of *A. hydrophila* YJ-1 to EPC cells (Figure 5). QS systems modulate bacterial adherence, it was proved that QS signaling plays an important role in cell attachment and even biofilm survival [25].

### 2.4. Assessment of AiiA_QSI-1_ Protection Against A. hydrophila Infection

**Figure 6:**
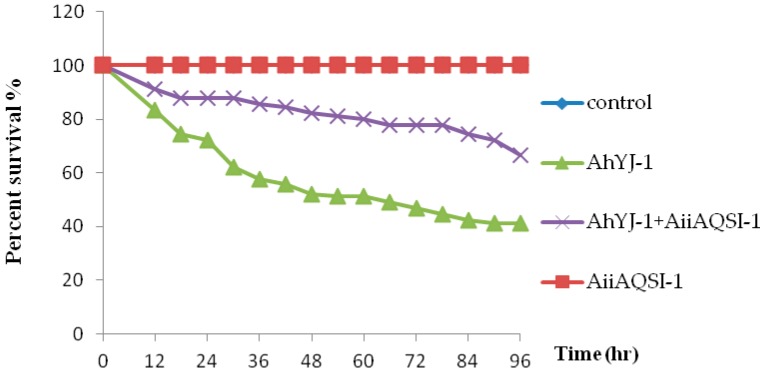
Percent survival of *Crucian carp* supplement with/without AiiA_QSI-1_ after *Aeromonas hydrophila* YJ-1 infection.

The results of lethal dose 50% (LD_50_) evaluation showed that strain YJ-1 to Crucian carp was 1.2 × 10^6^ Colony-Forming Units (CFU)/fish. Artificial challenge experiments were performed to detect the protection effect of AiiA_QSI-1_ against *A. hydrophila* YJ-1 infection in *Crucian carp*. Survival data of fish within 96 hrs for the four groups (*A. hydrophila* YJ-1 + AiiA_QSI-1_, *A. hydrophila* YJ-1, AiiA_QSI-1_, and control) are shown in Figure 6. No dead fish were observed in the AiiA_QSI-1_ and control groups, indicating that AiiA_QSI-1_ is not toxic for *Crucian Carp*. In contrast, fish infected with *A. hydrophila* YJ-1 increased mortality rate, but AiiA_QSI-1_ significantly attenuated the *A. hydrophila* YJ-1 infection. Compared with group *A. hydrophila* YJ-1, the survival percent of group feeding with AiiA_QSI-1_ was significantly higher than the commercial diet group infected with AhYJ-1, increased about 25.6% at day 4. The dead fishes presented hemorrhagic septicemia symptoms, and bacteria isolated from dead fishes’ organs (liver, spleen, and kidney) and ascites were identified as *A. hydrophila* by selective medium and biochemical tests as described by Palumbo et al. [26].

Biofilm formation has been associated with antibiotic resistance in a wide variety of bacteria including *A. hydrophila* [16,17]. In some organisms, notably *Pseudomonas aeruginosa*, biofilm-mediated antibiotic tolerance is regulated by QS in that QS mutants as well as QS inhibition can increase biofilm susceptibility to antibiotics [7]. Thus, the QQ demonstrated by AiiA_QSI-1_ (this study) and other QQ agents [21,22,27,28,29] has the added potential benefit of reducing antibiotic resistance in *A. hydrophila* biofilms as well as reducing *A. hydrophila* virulence. There are several issues that would need to be addressed in pilot-scale studies. In the first instance, the economic aspect of QQ production and its influence on commercial fish production would need to be assessed in both short-term (single harvest) and longer-term (multiple fish harvests). Secondly, longer term studies would enable a thorough assessment of any potential toxic side-effects of QQ treatments on fish. A third benefit of a pilot-scale study would be to investigate whether there might be an onset of resistance to quorum-disrupting treatments. The potential for resistance to quorum disruption has been proposed by Garcia-Contreras et al. but to our knowledge has not been thoroughly investigated [30]. A pilot-scale study would allow these important questions to be addressed.

Our results shown that oral administration of AiiA_QSI-1_ via feed supplementation can significantly attenuate *A. hydrophila* infection in *Crucian Carp*. The protection effects of recombinant AHLs degrading proteins against *A. hydrophila* were reported by other investigators. The AHL-lactonase AiiAB546 from *Bacillus* sp. B546 was expressed extracellularly in *Pichia pastoris*, and could decrease the mortality rate in common carp when it was co-injected with *A. hydrophila* [27]. AiiAAI96, from *Bacillus* sp. AI96 significantly attenuated *A. hydrophila* infection in zebra fish when administered orally as feed supplementation [28]. Liu et al. reported that, lactonase AIO6 supplemented to tilapia was able to prevent the *A. hydrophila* infection [29]. In addition to testing the impact of cloned and expressed QQ on *A. hydrophila* invasiveness (Figure 5) and biofilm formation (Figure 4E), our study is in agreement with previous investigations that show QQ causing reduced virulence and enhanced survival of economically important organisms, such as *Crucian carp* associated with aquaculture. Given the apparent lack of toxicity of QQ-supplemented feed on *Crucian carp* survival (Figure 6), we conclude that QQ-containing feed supplements should be explored for commercial use in aquaculture.

## 3. Materials and Methods

### 3.1. Bacterial Strains and Culture Conditions

The bacterial strains used in this study were *Chromobacterium violaceum* 026 (CV026), *Bacillus* sp. strain QSI-1 [17], *Aeromonas hydrophila* YJ-1 [31] and *E. coli* strain BL21(DE3) (Promega, USA). *Bacillus* sp. strain QSI-1 and *E. coli* strain BL21(DE3) were cultured in Luria-Bertani (LB) broth medium (Beijing Aoboxing Bio-Tech CO., Ltd., China) at 150 rpm and 37 °C. *C. violaceum* 026 was cultured overnight in LB broth, at 150 rpm and 28 °C. *A. hydrophila* YJ-1 was cultured in LB broth, at 150 rpm and 30 °C for 18–24 h, and bacterial cells were washed with sterile physiological saline three times, and suspended in physiological saline as injection preparation. For long-term storage, bacterial strains were preserved at −70 °C in LB containing 15% (*v*/*v*^−1^) glycerol.

### 3.2. Cloning, Expression, and Purification of AiiA_QSI-1_ Protein

Total genomic DNA from *Bacillus* sp. strain QSI-1 was extracted using an Ezup Column Bacteria Genomic DNA Purification Kit (Sangon Biotech, Shanghai, China) and used as template for amplification of *aiiA* gene by PCR. The forward primer was *aiiA*-F (5′-GACACCATATGACCGTCAAGAAGCTGTAC-3′) and reverse primer was *aiiA*-R (5′-GTCTCCTCGAGGATGTATTCCGGGAATAC-3′) with a product size of 773 bp bearing Nde I and Xho I recognition sites at each of the end sites. The recovered *aiiA* gene was double digested by Nde I and Xho I enzymes (New England Biolabs, Ipswich, MA, USA) and ligated into pET-30a vector (New England Biolabs, Ipswich, MA, USA) previously digested with the same restriction enzymes. The recombinant pET-30a-*aiiA_QSI-1_* vector was then transformed into *E. coli* BL21(DE3) (Promega, Madison WI,, USA). The procedure was carried out according to the method of Fan et al. [32]. After inducible expression with 0.5 mM IPTG at 37 °C for 4 h, cells were harvested and disrupted by sonication. The protein of interest was purified by a Ni-NTA His∙Bind column. The molecular mass of the expressed protein was determined by sodium dodecyl sulfate-polyacrylamide gel electrophoresis (SDS-PAGE). Proteins were stained with Coomassie brilliant blue G-250. The protein concentration was detected utilizing the Bradford protein assay (BioRad, Richmond, CA, USA) with bovine serum albumin (BSA) as a standard according to the manufacturer’s instructions [33]. Purified AiiA_QSI-1_ protein was suspended in PBS buffer (pH 7.3) and stored at −20 °C before use.

### 3.3. AHL-Lactonase Activity Bioassay

The AHL-lactonase activity of AiiA_QSI-1_ was quantified using *Chromobacterium violaceum* CV026 as an AHL-reporter strain [34]. Briefly, C6-HSL (*N*-hexanoyl homoserine lactone) as the substrate, different volumes of AiiA_QSI-1_ were mixed with 10 µL C6-HSL (10 μM) and then dropped into the well on the LB agar plate, which was previously spread with 50 µL of overnight cultured CV026 culture. The LB plates were incubated at 30 °C for 24 h. Subsequently, the diameter of purple violacein zones appearing on the plates was measured. The residual C6-HSL concentration can be extrapolated based on a standard curve relating the C6-HSL concentration with the diameter of the violacein zone induced by CV026 culture.

### 3.4. Effect of AiiA_QSI-1_ on the Motility, Production of Virulence Factors and Biofilm Formation in Aeromonas Hydrophila YJ-1

#### 3.4.1. Swimming Motility Assay LB Medium with 0.3% (*w*/*v*)

Difco Bacto Agar was used to characterize the swimming motility of *A. hydrophila* YJ-1 as previously described [35]. The overnight cultures of *A. hydrophila* YJ-1 grown in LB broth were briefly stabbed into agar plates with or without AiiA_QSI-1_. The swimming agar plates were incubated at 30 °C for 16–18 h and then motility assessed by measuring the migration of bacteria from the initial inoculation point. The swimming motility was detected in triplicate.

#### 3.4.2. Measurement of the Proteolytic and Hemolytic Activity

Overnight culture of *A. hydrophila* YJ-1 treated or untreated with AiiA_QSI-1_ was centrifuged at 12,000× *g* for 15 min. The supernatant was filtered by using 0.45-μm sterilized film. The supernatant was stored at −20 °C for extracellular proteolytic and hemolytic activity assay.

The extracellular proteolytic activity was detected using 1.0% skim milk plate as described by the method of Nicodème et al. [36] and Cole et al. [37]. 10 mL 10.0% (*w*/*v*) milk was added to 90 mL Nutrient agar (containing 1.8 g nutrient broth medium and 1.5% agar), following autoclaving, when the temperature dropped to 50 °C. The cell-free supernatant (100 μL) was added to the well and incubated at 37 °C for 18–24 h. The extracellular proteolytic activity was determined by measuring the diameter of the transparent zone around the hole in the milk plates. Each measurement was done in triplicate.

Hemolytic activity test was performed using 5% (*v*/*v*) sheep blood in a nutrient agar plate [38]. The cell-free supernatant (100 μL) was added to the well and incubated at 37 °C for 18–24 h. The β-hemolytic activity was determined by measuring the diameter of the transparent zone around the hole in the sheep blood plates. Each measurement was done in triplicate.

#### 3.4.3. Growth Curve Assay

We referenced previous method described by Zhou et al. to measure the influence of AiiAQSI-1 on the growth of *A. hydrophila* YJ-1 [39]. Bacterial cells were grown in LB medium to a final optical density (OD_600_) of 0.2. Then the culture was transferred to a 250-mL flask containing 50-mL LB medium and cultured at 28 °C with 150-rpm shaking. The OD_600_ value was determined and the growth curve was recorded. This experiment was repeated three times.

#### 3.4.4. Crystal Violet (CV) Biofilm Assay

The inhibition of AiiA_QSI-1_ on the biofilm formation by *A. hydrophila* YJ-1 was tested using crystal violet staining assay as described by Coffey and Anderson [40]. In brief, an overnight inoculum was diluted 100 times in LB medium and 100 μL of each dilution was inoculated into the wells of 96-well microtiter polystyrene plates (Nunc-Immuno MaxiSorp; Nunc, Rochester, NY, USA) in triplicate. The microtiter plates were incubated at 28 °C for 36 h to allow biofilm formation. Planktonic bacteria was detected by measuring the optical density (OD) at 600 nm with a spectrophotometer (Shanghai Selon Scientific Instrument Co., Ltd., Shanghai, China). After incubation, the microtiter plates were rinsed thoroughly with distilled water and the remaining cells were stained with 150 μL 0.5% crystal violet solution for 20 min at room temperature. Excess crystal violet solution was removed, and the wells were washed three more times. The crystal violet-stained biofilm was then solubilized by the addition of 100% ethanol, and then the biofilm quantification was performed at 595 nm as previously described by Hraiech et al. [41]. The experiment was repeated independently three times.

### 3.5. Effect of AiiA_QSI-1_ on Bacterial Adhesion and Invasion to EPC Cells

Adherence and internalization assays were detected using the method described by Yang et al. [42]. Briefly, Epithelioma Papulosum Cyprini (EPC) cells were incubated in 24-well tissue culture plates, allowing a confluent monolayer to form, and then infected by *A. hydrophila* YJ-1 at a multiplicity of infection of 10. After incubation at 28 °C for 3 h, the cells were washed with PBS to detect the bacterial adhesion. To measure the number of bacteria adhering and invasion into the mono-layers, the cells were lysed with 1% Triton X-100 and harvested, after serial dilution, the lysate was then spread on LB agar plates using a glass spreader and incubated at 28 °C overnight for bacteria counting. For internalization assay, the mono-layers were incubated for an additional 2 h in MEM containing 200 μg mL^−1^ gentamycin (*A. hydrophila* YJ-1 is gentamycin sensitive) before being lysed and harvested and then bacterial numbers quantified by plate counting. The adhesion and internalization rates were calculated from the mean of at least two wells in quadruplicate experiments.

### 3.6. Effect of AiiA_QSI-1_ on the Protection Against Aeromonas Hydrophila Infection

*Crucian carps* (*Carassius auratus gibelio*; 50.0 ± 2.5 g and 12.0 ± 1.5 cm) were obtained from an aquaculture farm in Nanjing, Jiangsu Province, China. Prior to experimentation, 600 fishes were acclimatized in 12 aquariums (100 L) for 10 days, at a temperature of 25 ± 1.0 °C, dissolved oxygen >5 mg/L, and given commercial feed twice each day at 08:30 and 17:30. The commercial feed was supplied by Huaian Tongwei Co. Ltd., Huaian, China, and contained 47% fishmeal, 24% soybean meal, 24% wheat flour, 2% soybean oil, 3% premix, and no antibiotics (42.0% crude protein and 7.29% crude lipid). For 50% lethal dose (LD50) determinations, seven groups of 10 *Crucian carps* were intraperitoneally injected with 0.02 mL of serially tenfold diluted bacterial suspensions containing 10^1^–10^8^ CFU. A control group was injected intraperitoneally with 0.02 mL sterile PBS only. The fish were monitored at 25 ± 1.0 °C for mortality for 7 days. During this period, activity and behavior of fish were recorded daily, and LD_50_ were calculated by the method of Reed and Muench [43]. For the preparation of AiiA_QSI-1_ containing diets, the AiiA_QSI-1_ protein was suspended in saline solution and mixed with basal diet at final concentration 0.5g AiiA_QSI-1_ per kilogram. The mixed feed was oven-dried at 45 °C for 4 h and stored at −20 °C in sealed plastic Ziploc bags until used. Fishes were divided randomly into four groups with three replicates, each group with 30 fishes: groups A and C were fed with the commercial diet, and groups B and D were fed with the commercial diet supplemented with AiiA_QSI-1_ protein. After 10 days of feeding the fishes, groups C and D were intraperitoneally injected with 200 μL of *A. hydrophila* YJ-1 suspension containing 6.0 × 10^6^ cells (5 × LD_50_), and groups A and B were administered 200 μL sterile physiological saline. The fish survival was recorded every day during the 4-days of experimental period, and dead fishes were removed for bacteriological inspection [27]. For identification of *A. hydrophila*, Starch-Ampicillin agar was used as selective medium as described by Palumbo et al. [26]. The animal experiments were carried out under the instruction and supervision of the Ethical Committee for Animal Experiments of China Pharmaceutical University (Nanjing, China). All the procedures abided by the guidelines of laboratory animal welfare ethical review and regulations for the administration of affairs concerning experimental animals in China.

## 4. Statistical Analysis

All tests were carried out in triplicate and data were presented as the mean values. Analysis of variance was conducted and differences between the mean values were tested for significance using one-way ANOVA with Tukey test correction on the SPSS Statistics 20.0, Origin Pro 8.0. Differences with a *p* < 0.05 were considered statistically significant.

## 5. Conclusions

In conclusion, a recombinant lactonase enzyme, AiiA_QSI-1_ could significantly reduce the whole extracellular protease production, and hemolytic activity in *Aeromonas hydrophila* YJ-1. It also displayed an inhibitory activity on the swimming motility, adherence to epithelial cells and the biofilm formation. The oral administration of AiiA_QSI-1_ supplement in feed significantly attenuated *Aeromonas hydrophila* YJ-1 infection in carp. Our results indicate that the AiiA_QSI-1_ represents a promising candidate to use in pilot-scale tests as a feed-based therapeutic agent for aquaculture and provides a viable alternative to antibiotics against *Aeromonas* diseases in aquaculture. Pilot scale tests would enable a more rigorous investigation of the longer-term potential of this antibiotic-alternative disease prevention strategy in aquaculture.

## Figures and Tables

**Table 1:** Diameters of transparent zone for proteolytic activity and hemolytic activity.

	AiiA _QSI-1_ (mg/mL)/Diameter (cm)			
	0	0.0383	0.0766	0.1149
Protease	2.46 ± 0.0794	1.7367 ± 0.0651	1.17 ± 0.0529	0.8233 ± 0.095
Hemolysin	1.8567 ± 0.0321	0.97 ± 0.1054	0.7067 ± 0.0737	0.5767 ± 0.0404
